# Complex Interplay between Autophagy and Oxidative Stress in the Development of Endometriosis

**DOI:** 10.3390/antiox11122484

**Published:** 2022-12-17

**Authors:** Ramona D’Amico, Daniela Impellizzeri, Marika Cordaro, Rosalba Siracusa, Livia Interdonato, Ylenia Marino, Rosalia Crupi, Enrico Gugliandolo, Francesco Macrì, Davide Di Paola, Alessio Filippo Peritore, Roberta Fusco, Salvatore Cuzzocrea, Rosanna Di Paola

**Affiliations:** 1 Department of Chemical, Biological, Pharmaceutical and Environmental Sciences, University of Messina, Viale Ferdinando Stagno D’Alcontres, n 31, 98166 Messina, Italy; 2 Department of Biomedical, Dental and Morphological and Functional Imaging, University of Messina, Via Consolare Valeria, 98125 Messina, Italy; 3 Department of Veterinary Sciences, University of Messina, Viale Annunzita, 98168 Messina, Italy

**Keywords:** endometriosis, autophagy, mitophagy, oxidative stress, apoptosis

## Abstract

Endometriosis (Endo) is a chronic gynecological disease. This paper aimed to evaluate the modulation of autophagy, oxidative stress and apoptosis with Açai Berries in a rat model of endometriosis. Endometriosis was induced with an intraperitoneal injection of minced uterus tissue from a donor rat into a recipient one. The abdominal high-frequency ultrasound (hfUS) analysis was performed at 7 and 14 days from the endometriosis induction to evaluate the growth of the lesion during the experiment. Seven days from the induction, once the lesions were implanted, an Açai Berry was administered daily by gavage for the next seven days. At the end of the experiment, the hfUS analysis showed a reduced lesion diameter in animals given the Açai Berry. A macroscopical and histological analysis confirmed this result. From the molecular point of view, Western blot analyses were conducted to evaluate the autophagy induction. Samples collected from the Endo group showed impaired autophagy, while the Açai Berry administration inhibited PI3K and AKT and ERK1/2 phosphorylation and promoted autophagy by inactivating mTOR. Additionally, Açai Berry administration dephosphorylated ATG1, promoting the activity of the ATG1/ULK1 complex that recruited Ambra1/Beclin1 and Atg9 to promote autophagosome nucleation and LC3II expression. Açai Berry administration also restored mitophagy, which increased Parkin cytosolic expression. The Açai Berry increased the expression of NRF2 in the nucleus and the expression of its downstream antioxidant proteins as NQO-1 and HO-1, thereby restoring the oxidative imbalance. It also restored the impaired apoptotic pathway by reducing BCL-2 and increasing BAX expression. This result was also confirmed by the TUNEL assay. Overall, our results displayed that Açai Berry administration was able to modulate autophagy, oxidative stress and apoptosis during endometriosis.

## 1. Introduction

Endometriosis is a chronic disease of the endometrium [1,2,3]. The abnormal infiltration and growth of stromal cells and endometrial epithelial cells causes the formation of masses and nodules [2,4]. These lesions induce dysmenorrhea, chronic pelvic pain and infertility [5,6]. Actual endometriosis affects 30 to 50% of women in menopause and 15% of women of reproductive age [7]. The most accepted theories that explain the invasion and implantation of endometrial tissue are the ectopic presence of endometrial stem cells [8], retrograde menstrual reflux [9] and defects in the immune system [10].

Evidence from the literature shows a dysregulated antioxidant/pro-oxidant balance and an increased proinflammatory microenvironment in endometrial lesions [11]. Recently, increasing importance has been assigned to the autophagic pathway in the induction of the endometriosis [12,13,14]. It is the major constitutive pathway for the degradation of cytoplasmic organelles and long-lived proteins in eukaryotic cells [15,16]. This catabolic pathway mediates both the targeted and nonspecific sequestration of macromolecules and cellular organelles, promoting the recycling of useful metabolites and permitting the degradation of cellular constituents in lysosomes [17]. Autophagy can be deleterious to the cell when its activation is too extensive, and it can induce cell death. Differently, a basal autophagic response acts as a survival and housekeeping mechanism that maintains cellular homeostasis in physiological conditions and contributes to overcoming the stressful conditions induced by both extracellular and intracellular stimuli, including reduced nutrient supply, hypoxia, invasion of microorganisms, oxidative stress and therapeutic stress [18,19,20]. Autophagy is also responsible for the elimination of damaged or aged organelles. Mitophagy or mitochondrial autophagy is the selective mechanism to remove the dysfunctional mitochondria [21]. Indeed, autophagy shows a key role in inducing cell death by promoting caspase-dependent apoptosis in homeostatic conditions [22,23]. For instance, the autophagic machine has important roles in the process of differentiation, growth, cell immunity, tissue remodeling and environmental adaptation [24,25]. In normal endometrial cells, the induction of autophagy exercises proapoptotic effects [26]. Meanwhile, ectopic endometrial cells showed a reduced autophagic pathway compared with the normal endometrium [27]. The autophagic pathway was impaired in the endometriotic-like lesions of the mice, and the autophagic markers were altered as compared to the control [28]. Strongly associated with autophagy, apoptosis is one of the main impaired pathways during endometriosis because it contributes to the survival of the ectopic cells and the growth of the lesions [29].

Thus, several papers described that autophagic and apoptotic activators would reduce the development of this pathology by reducing the growth of the lesions [30]. Recently, increased interest has been developed for the nutritional properties and medicinal uses of Açai Berries [31,32,33]. They are an Amazonian fruit produced by the Euterpe oleracea palm. For millennia, it has been used by Indians as a natural mixture to treat many diseases [34,35,36,37]. Açai Berries, in fact, contain many biologically active phytochemicals including quercetin, luteolin, delphinidin, cyanidin, malvidin and pelargonidin [38]. Several studies report that Açai Berries have neuroprotective, anti-inflammatory and antioxidant properties [38,39,40,41,42]. Recently, the modulation of the autophagic pathway by the Açai Berry supplementation was reported [33]. However, more data are required to confirm the beneficial effect of Açai Berries. In this paper, we employed a well-consolidated endometriosis model to investigate the effects of Açai Berry administration and the molecular pathway involved.

## 2. Materials and Methods

### 2.1. Animals

Female Sprague–Dawley rats were employed in this study. The University of Messina Review Board for animal care (OPBA) approved this study. All animal experiments complied with the new Italian and EU regulations (D.Lgs 2014/26, EU Directive 2010/63).

### 2.2. Experimental Protocol

Rats were randomly distributed into two groups, donor or recipient, and endometriosis was induced as already described [43]. To establish similar estrogen levels among the rats, donor animals were administered 10 IU pregnant mare serum gonadotropin. After 41 h, the rats were euthanized and the uterus was removed. Tissue was minced with scissors in a 1.5 mL centrifuge tube containing PBS. Tissue from all the donors was pooled, and the equivalent of one uterus/500 uL of PBS was intraperitoneally injected along the midventral line of the recipient animals. Endometriosis was allowed to develop for seven days.

The success rate for the lesion development was 70% [44].

### 2.3. Experimental Groups

The rats were randomized and assigned to the following groups (*n* = 12):

(1) Endo group: rats were subjected to experimental endometriosis and vehicle (saline) was administered by a gavage on the 7th day and for the next 7 days.
(2) Endo + Açai Berry group: rats were subjected to experimental endometriosis as described and an Açai Berry (200 mg/kg) was orally administered on the 7th day and for the next 7 days.
(3) Sham group: rats were injected intraperitoneally with 500 uL of PBS instead of endometrial tissue, and a vehicle (saline) was administered on the 7th day and for the next 7 days.

The Açai Berry dose was based on previous studies [45]. In order to evaluate the effect of the Açai Berry administration on the endometriotic-like lesions, the rats were sacrificed 14 days after the induction. Thereafter, a laparotomy was performed to collect the endometriotic implants for further analyses.

### 2.4. Abdominal High-Frequency Ultrasound

Ultrasonographic exams were performed using the Esaote MYLAB OMEGA VET on anesthetized rats (2% isoflurane) positioned in dorsal recumbency. An abdominal B-mode was performed with a High Frequency Linear array (4–15 MHz) transducer [46]. Longitudinal and transverse scanning planes were employed for the evaluation of different abdominal structures.

### 2.5. Histological Examination

Endometriotic lesions were fixed in a formaldehyde solution and were embedded in Paraplast [47,48]. Tissue slides were stained with H&E and were evaluated using a Leica DM6 microscope (Leica Microsystems SpA, Milan, Italy). A histological analysis was performed using a double-blind procedure. Histopathological scores were assigned according to the formula P (persistence of epithelial cells in the explants) × I (intensity of glands), as already described [30]. The lesion volume was calculated according to the formula V = (length × width2) × 0.5 [49].

### 2.6. Terminal Deoxynucleotidyl Nick-End Labeling (TUNEL) Assay

Apoptosis was analyzed with a TUNEL assay using an in situ cell death detection kit (Roche 11684795910) [50,51,52].

### 2.7. Western Blot Analysis

Western blots were performed as already described to obtain either cytosolic and mitochondrial [53] or cytosolic and nuclear [54] protein fractions. The specific primary antibodies anti-Beclin (sc-48381, Heidelberg, Germany), anti-mTOR (Cell Signaling, 2972, Milan, Italy), anti-p-mTOR (sc-293089, Heidelberg, Germany), anti-p-AKT (sc-293125, Heidelberg, Germany), anti-AKT (Invitrogen AHO1112, London UK), anti-LC3 II (Sigma Aldrich, ABC232, Milan, Italy), anti-AMBRA1 (Abcam, Ab69501, Cambrige, UK), anti-IP3K (sc-1637), anti-BCL-2 (sc-7382, Heidelberg, Germany), anti-PARKIN (sc-32282, Heidelberg, Germany), anti-BAX (sc-7480, Heidelberg, Germany), anti-NQO1 (sc-32793, Heidelberg, Germany), anti-HO1 (sc-136960, Heidelberg, Germany), anti-PINK1 (sc-517353, Heidelberg, Germany), anti-NRF2 (sc-365949, Heidelberg, Germany), anti-p-ERK (sc-7383, Heidelberg, Germany), anti-ATG9 (cell signaling 13509, Milan, Italy), anti-ERK (sc-514302, Heidelberg, Germany), anti-p-ATG1 (Bioss, BS-3464R, Cambrige, UK) and anti-ATG1 (Sigma, A7481, Cambrige, UK) were mixed in a 5% *w/v* nonfat dried milk solution and were incubated at 4 °C overnight. Blots were incubated with a peroxidase-conjugated goat antirabbit IgG (Jackson Immuno Research) or a peroxidase-conjugated bovine antimouse IgG secondary antibody for 1 h at room temperature [55,56]. To confirm the equal amounts of protein, filters were also incubated with the antibody against β-ACTIN (sc-47778), COXIV (ab14744) and HISTONE 3 (ab1791). Signals were detected with an enhanced chemiluminescence detection system reagent (Super-Signal West Pico Chemiluminescent Substrate) [57,58]. The relative expression of the protein bands was quantified using densitometry with Bio-Rad ChemiDoc XRS software, #1708265 [59]. Images of the blot signals were imported to analysis software (Image Quant TL, Amersham Biosciences, Freiburg, Germany, v2003) [60,61].

### 2.8. Biochemical Analysis

Lipid peroxidation was evaluated with the TBARS test by reading the MDA levels at 535 nm [54,62]. SOD activity was evaluated as already described [47,63] and is expressed as U/g protein [64]. GSH levels were determined using a microplate reader at 412 nm [65,66].

### 2.9. Statistical Analysis

All the values are expressed as mean ± standard error of the mean of N observations. The results were analyzed with a t-test when comparing the two groups, and we used the t-test and the Kolmogorov–Smirnov test to analyze the normal distribution of the data (Prism 8 for macOS version 8.2.1 (279)). A *p*-value of less than 0.05 was considered significant. * *p* < 0.05 vs. Endo, ** *p* < 0.01 vs. Endo, *** *p* < 0.001 vs. Endo.

## 3. Results

### 3.1. Effect of Açai Berry on Endometriotic-Like Lesions Development

**Figure 1:**
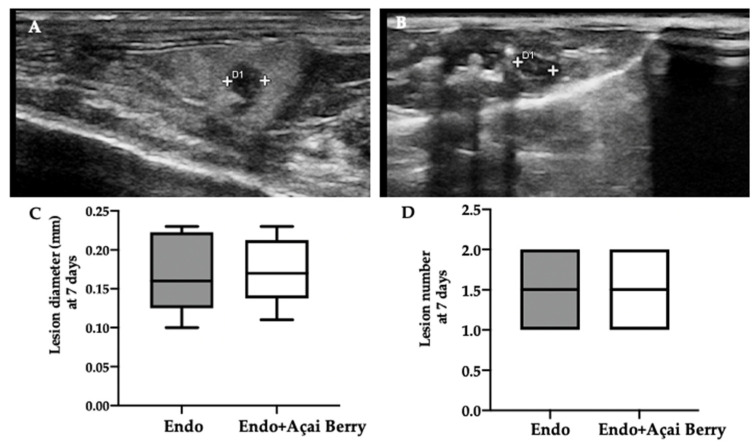

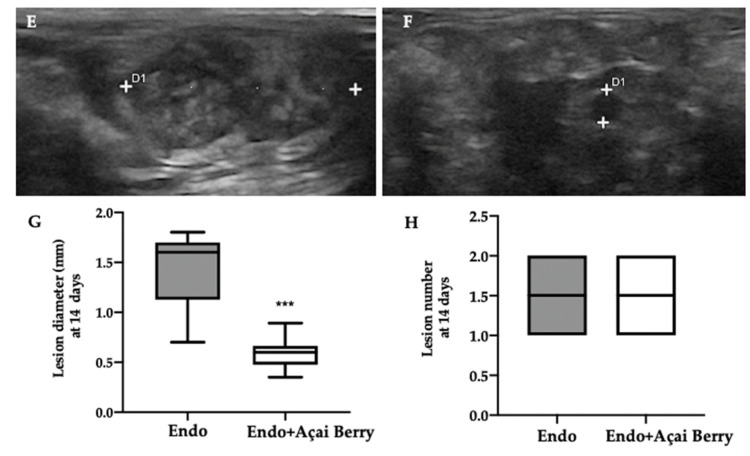
Analysis of endometriotic-like lesions development: High-frequency ultrasound analysis (hfUS) at 7 days from the endometriosis induction: Endo (**A**), Endo + Açai Berry (**B**), lesion diameter (**C**) and lesion number (**D**). hfUS analysis at 14 days from the endometriosis induction: Endo (**E**), Endo + Açai Berry (**F**), lesion diameter (**G**) and lesion number (**H**). A *p*-value of less than 0.05 was considered significant. *** *p* < 0.001 vs. Endo.

Ultrasonographic exams were employed to monitor the development of the pathology at seven and fourteen days from the induction. A pelvic ultrasound showed endometriotic-like lesions in the inner surface of the peritoneal cavity in both groups at seven days from the induction (Figure 1A,B). This analysis was conducted to control the establishment of the pathology before the Açai Berry administration. No differences were detected in diameter (Figure 1C) and lesions number (Figure 1D). After this control was applied, the Açai Berry was administered for the next 7 days. Fourteen days from the induction, the ultrasonographic exams showed that the Endo group had an increased lesion diameter (Figure 1E,G) as compared to the Endo + Açai Berry group (Figure 1F,G). The same number of lesions was detected in both groups (Figure 1H).

### 3.2. Effect of BS Administration on Macroscopic and Histological Analysis

**Figure 2:**
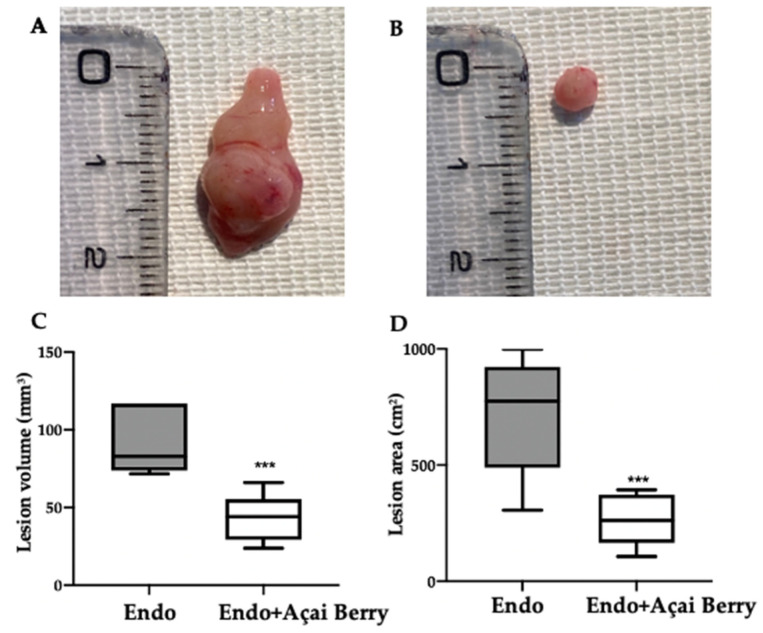

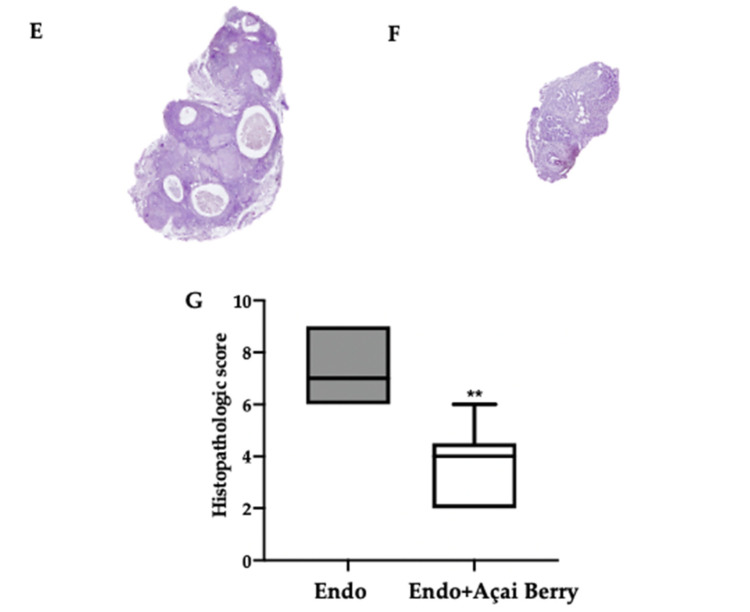
Analysis of Açai Berry administration on macroscopical and histological analysis: Macroscopic analysis: Endo (**A**), Endo + Açai Berry (**B**), lesion area (**C**) and lesion volume (**D**). Histological analysis: Endo (**E**), Endo + Açai Berry (**F**) and histopathologic score (**G**). A *p*-value of less than 0.05 was considered significant. ** *p* < 0.01 vs. Endo, *** *p* < 0.001 vs. Endo.

Fourteen days after the rats developed endometriosis, an induction laparotomy was performed in both groups and lesions were harvested. The macroscopic analysis (Figure 2A,B) was in line with the ultrasonographic exams. The endometriosis lesions collected from the Endo group showed a higher volume (Figure 2C) and area (Figure 2D) that than collected from the Endo + Açai Berry group. The histopathological analysis showed that the Açai Berry administration changed the lesion morphology. Lesions harvested from the Endo group presented characteristic glands and stroma (Figure 2E,G), while the Açai Berry administration reduced the histopathological score (Figure 2F,G).

### 3.3. Effect of BS Administration on Autophagy Inhibition Induced by Endometriosis

**Figure 3:**
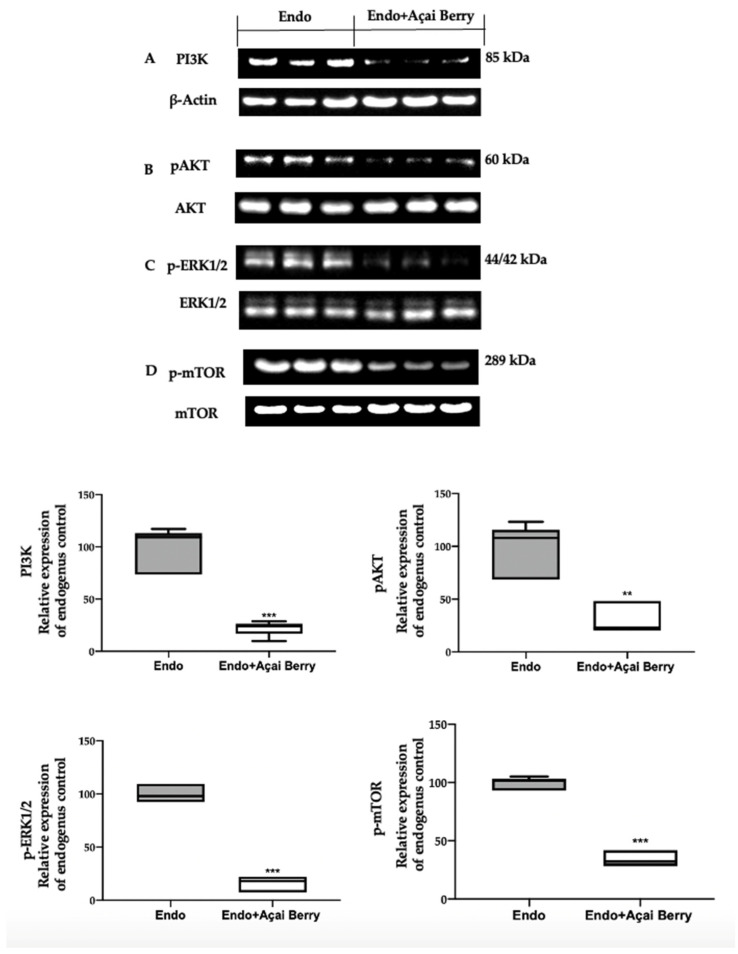
Analysis of Açai Berry administration on PI3K/AKT/ERK1/2 pathways. Western blot analysis of PI3K (**A**), pAKT (**B**), p-ERK1/2 (**C**) and p-mTOR (**D**) expression. A *p*-value of less than 0.05 was considered significant. ** *p* < 0.01 vs. Endo, *** *p* < 0.001 vs. Endo.

A Western blot analysis was employed to evaluate the modulation of the autophagic pathway induced by the Açai Berry administration. Samples collected from the Endo group showed an elevated PI3K expression (Figure 3A) and an increased phosphorylation of AKT (Figure 3B), ERK (Figure 3C) and mTOR (Figure 3D). Differently, in the samples harvested from the Endo + Açai Berry group, the PI3K expression (Figure 3A) decreased, as did the pAKT (Figure 3B), p-ERK1/2 (Figure 3C) and the p-mTOR (Figure 3D) levels.

**Figure 4:**
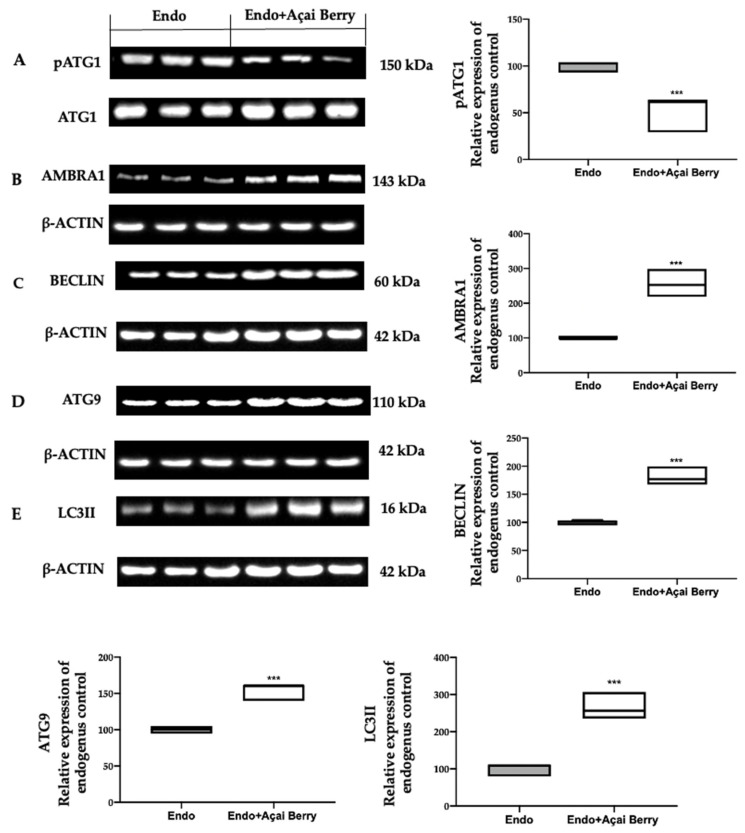
Analysis of Açai Berry administration on the autophagosome formation. Western blot analysis of pATG1 (**A**), AMBRA1 (**B**), BECLIN (**C**), ATG9 (**D**) and LC3II (**E**) expression. A *p*-value of less than 0.05 was considered significant. *** *p* < 0.001 vs. Endo.

To further evaluate the autophagosome formation, we checked the phosphorylation of the ATG1/ULK1 complex and the expression of the downstream proteins. The samples collected from the Endo group showed elevated ATG1 phosphorylation (Figure 4A) and low AMBRA1 (Figure 4B), BECLIN (Figure 4C), ATG9 (Figure 4D) and LC3II (Figure 4E) expressions. The Açai Berry administration reduced Atg1 phosphorylation (Figure 4A) and increased the expression of AMBRA1 (Figure 4B), BECLIN (Figure 4C), ATG9 (Figure 4D) and LC3II (Figure 4E).

### 3.4. Effect of BS Administration on Mitophagy Inhibition Induced by Endometriosis

**Figure 5:**
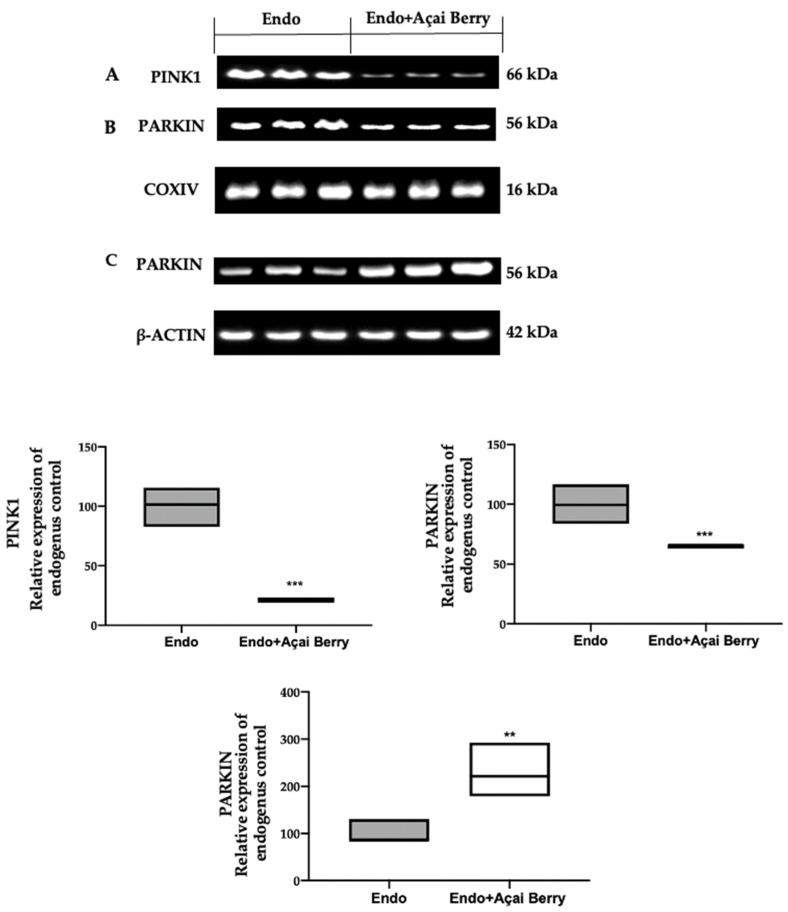
Analysis of Açai Berry administration on the Pink1 and Parkin expression. Western blot analysis of PINK1 (**A**) and PARKIN (**B**) mitochondrial expression and PARKIN (**C**) cytoplasmic expression. A *p*-value of less than 0.05 was considered significant. ** *p* < 0.01 vs. Endo, *** *p* < 0.001 vs. Endo.

To investigate mitophagy induction, we investigated the cytoplasmic and mitochondrial expression of PINK1 and PARKIN. The samples collected from the Endo group showed elevated PINK1 (Figure 5A) and PARKIN (Figure 5B) expressions in the mitochondria, while Parkin expression was reduced in the cytosol (Figure 5C). The Açai Berry administration reduced PINK1 (Figure 5A) and PARKIN (Figure 5B) mitochondrial expressions, while the PARKIN cytoplasmic expression was increased (Figure 5C).

### 3.5. Effect of BS Administration on Oxidative Imbalance Induced by Endometriosis

**Figure 6:**
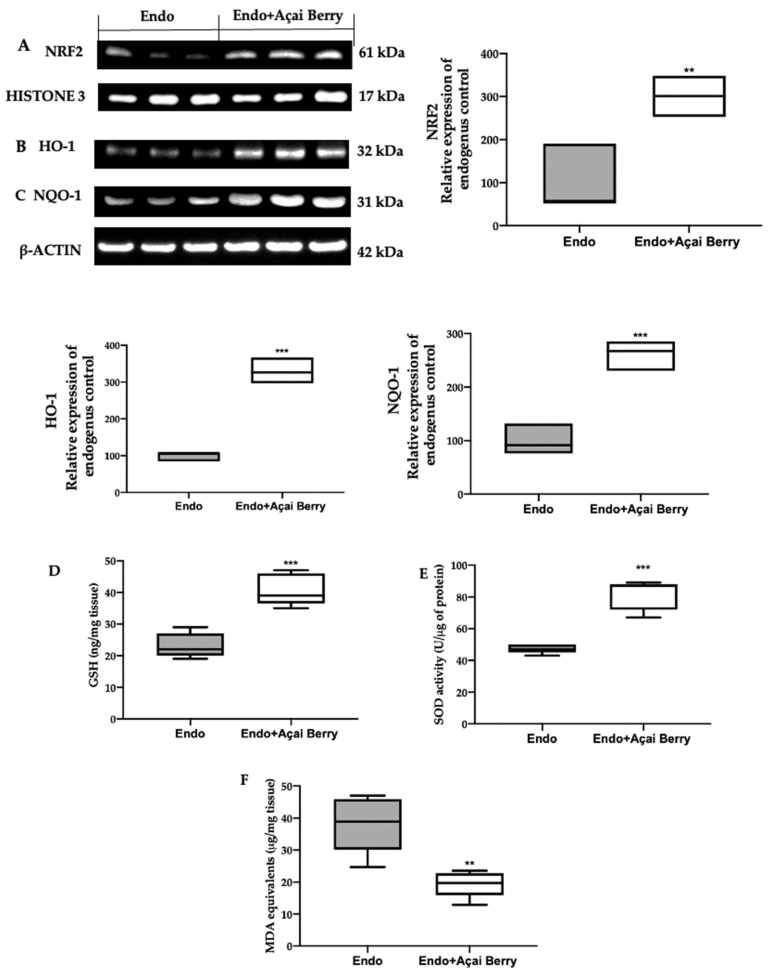
Analysis of Açai Berry administration on prooxidative alterations. Western blot analysis of NRF2 (**A**) nuclear expression, HO-1 (**B**) and NQO-1 (**C**) cytosolic expression, GSH levels (**D**), SOD activity (**E**) and MDA (**F**) levels. A *p*-value of less than 0.05 was considered significant. ** *p* < 0.01 vs. Endo, *** *p* < 0.001 vs. Endo.

In order to evaluate the oxidative alterations, the NRF2 pathway was examined. A Western blot analysis showed a low nuclear NRF2 expression (Figure 6A) and low cytosolic HO-1 (Figure 6B) and NQO-1 (Figure 6C) expression in the samples collected from the Endo group. Conversely, the Açai Berry administration increased the NRF2 nuclear expression (Figure 6A) and the cytosolic expression of the downstream proteins (Figure 6B,C).

The lesions collected from the Endo group also showed low GSH levels (Figure 6D) and SOD (Figure 6E) activity, while lipid peroxidation was found to be elevated (Figure 6F). The Açai Berry administration increased the GSH levels (Figure 6D) and SOD activity (Figure 6E) and reduced lipid peroxidation (Figure 6F).

### 3.6. Effect of BS Administration on Apoptosis Inhibition Induced by Endometriosis

**Figure 7:**
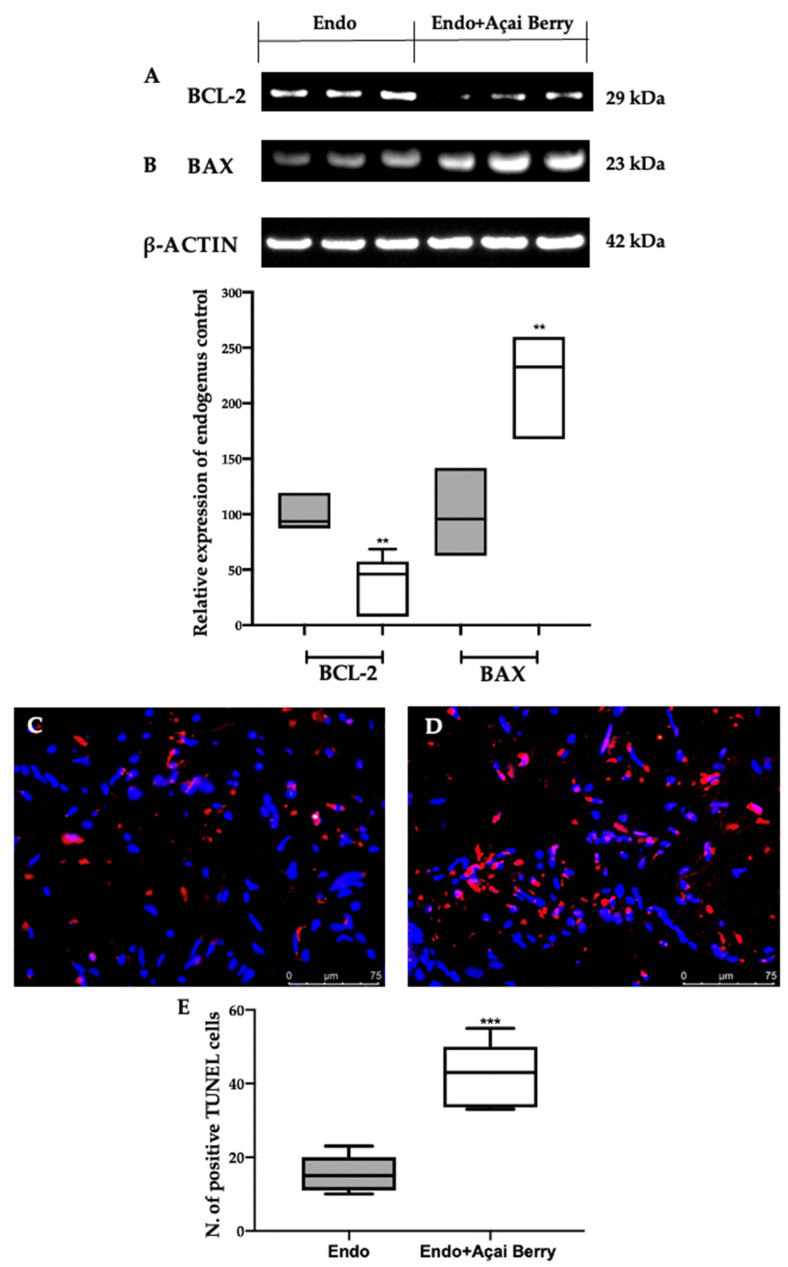
Analysis of Açai Berry administration on apoptosis impairment: Western blot analysis of BCL-2 (**A**) and BAX (**B**) expression. TUNEL assay: Endo (**C**), Endo + Açai Berry (**D**) and TUNEL-positive cells (**E**). A *p*-value of less than 0.05 was considered significant. ** *p* < 0.01 vs. Endo, *** *p* < 0.001 vs. Endo.

The samples collected from the Endo group showed an impaired apoptotic pathway (Figure 7). A Western blot analysis revealed an elevated BCL-2 (Figure 7A) and low BAX (Figure 7B) expression in the Endo group. The Endo + Açai Berry group showed a reduced BCL-2 (Figure 7A) and increased Bax (Figure 7B) expression. These results were confirmed with a TUNEL analysis where the number of TUNEL-positive cells strongly increased in the Endo + Açai Berry group (Figure 7D,E) as compared to the Endo group (Figure 7C,E).

## 4. Discussion

Endometriosis is a chronic disease with intricate molecular mechanisms. Açai Berries have important antioxidant, anti-inflammatory and neuroprotective proteins that have the ability to modulate the autophagic pathway in many diseases [38,39,40,41,42]. This paper aimed to evaluate the molecular mechanisms regulated by Açai Berry supplementations during endometriosis. The pathology was induced and monitored with an hfUS analysis. The Açai Berry supplementation reduced the lesion area, volume and diameter. From the molecular point of view, the endometrial microenvironment was characterized by dysregulated autophagic, oxidative balance and apoptotic pathways [33,67,68].

Many papers described that autophagy is suppressed in endometriotic cells by the PI3K/AKT/ERK1/2 pathways that positive regulate the expression of mTOR, which is the major modulator of autophagy [69,70,71,72,73]. Açai Berry administration inhibited PI3K and AKT and ERK1/2 phosphorylation and promoted autophagy by inactivating mTOR. mTOR has a central role in the regulation of cell growth and autophagy [74]. Inhibiting mTOR Açai Berry administration dephosphorylated ATG1, which promoted the activity of the ATG1/ULK1 complex. The ATG1/ULK1 complex recruits other proteins, including AMBRA1/BECLIN1 and ATG9, to promote autophagosome nucleation [75]. Additionally, Açai Berry supplementation increased the expression of AMBRA1 and BECLIN-1, which promotes the autophagic pathway transforming LC3I into its membrane-bound form of LC3-II [76]. These findings showed the role of the Açai Berry administration in the management of the autophagic pathway in endometriosis.

Although autophagy was initially considered a nonselective process, accumulating evidence has shown the presence of specific pathways for the degradation of damaged organelles [77,78]. Recent papers already described the role of mitochondrial autophagy in endometriosis [30]. Currently, PARKIN and PINK1 are the most well-studied proteins involved in the mechanism [79]. In the functional mitochondria PINK1, a serine/threonine kinase is continuously degraded by matrix-processing peptidase [80,81]. It is a sensor of organelle damage and an initiator of mitophagy [82]. In depolarized mitochondria, it accumulates in the outer mitochondrial membrane [83] and recruits PARKIN, a cytosolic E3 ubiquitin ligase [84]. PARKIN is a cytosolic protein that is recruited by PINK1 in impaired mitochondria. PARKIN-labeled mitochondria are polyubiquitinated [85]. The phospho-ubiquitin chain further recruits autophagy receptor proteins, triggering the formation of autophagosomes for degradation. The Açai Berry administration restored this organelle-specific autophagy by facilitating the removal of damaged mitochondria through mitophagy. Mitochondria are the major source of ROS; therefore, when the mitophagy function is impaired and unfunctional mitochondria are not removed properly, they increase ROS production, which aggravates tissue injury [86,87,88,89]. The endometrial microenvironment is, in fact, characterized by a dysregulated oxidative balance [24]. The NRF2 signaling controls the transactivation of several cytoprotective genes and is one on the most important regulatory pathways in defending cells from ROS [90]. Physiologically bound to its inhibitor KEAP1, NRF2 is usually polyubiquitinated by the E1 ligase complex and is degraded [33,91]. A dysregulated oxidative balance, which is characteristic of the disease, breaks the KEAP1-NRF2 link and allows for the NRF2 translocation into the nucleus [92]. Here it binds the antioxidant response elements (ARE), promoting the expression of cytoprotective genes with antioxidant and detoxifying roles [91]. The samples collected from the animals administered with Açai Berries showed an increased NRF2 nuclear expression as well the cytosolic expression of the cytoprotective proteins NQO-1 and HO-1. Well in line with the literature, where elevated lipid peroxidation and ROS were found in ectopic endometrium biopsies, Açai Berry supplementation restored the oxidative imbalance.

Increased oxidative stress and dysregulated autophagic pathways result in impaired apoptosis. It has been recently shown that the induction of autophagy has a proapoptotic effect on normal human endometrial cells [93]. While the overactivation of autophagy and apoptosis has been identified as damaging in many pathologies, during endometriosis they extern cytoprotective properties [94,95]. Indeed, they are tightly regulated by common signals [96,97,98]. Our results demonstrated the proapoptotic effect of the Açai Berry administration, which reduced the expression of the proapoptotic protein Bax and the antiapoptotic protein BCL2. This result was confirmed by the TUNEL assay, where apoptotic cells were identified by the terminal deoxynucleotidyl transferase (TdT)-mediated addition of labeled (X) de-oxyuridine triphosphate nucleotides (X-dUTPs) to the 3′-OH end of DNA strand breaks.

Overall, our result showed the role of the Açai Berry administration on the management of endometriosis, describing the modulation of the autophagy, oxidative stress and apoptosis.

## 5. Conclusions

Overall, this paper showed the key role of autophagy, oxidative stress and apoptosis in the development of endometriosis. Our results showed that Açai Berries modulate the PI3K/AKT/ERK1/2 pathways, thereby reducing the expression of mTOR and promoting the autophagy. Indeed, the Açai Berries facilitated the removal of damaged mitochondria through the activation of mitophagy and restored the oxidative imbalance and the impaired apoptosis.

## Data Availability

The data presented in this study are available upon request from the corresponding author.

## References

[1] Herington J.L., Bruner-Tran K.L., A Lucas J., Osteen K.G. (2011). Immune interactions in endometriosis. Expert Rev. Clin. Immunol..

[2] Giudice L.C. (2010). Clinical practice. Endometriosis. N. Engl. J. Med..

[3] Aghajanova L., Giudice L.C. (2011). Molecular Evidence for Differences in Endometrium in Severe Versus Mild Endometriosis. Reprod. Sci..

[4] Nisolle M., Donnez J. (1997). Peritoneal endometriosis, ovarian endometriosis, and adenomyotic nodules of the rectovaginal septum are three different entities. Fertil. Steril..

[5] Sepulcri R.d.P., do Amaral V.F. (2009). Depressive symptoms, anxiety, and quality of life in women with pelvic endometriosis. Eur. J. Obstet. Gynecol. Reprod. Biol..

[6] Gao X., Outley J., Botteman M., Spalding J., Simon J.A., Pashos C.L. (2006). Economic burden of endometriosis. Fertil. Steril..

[7] Fourquet J., Gao X., Zavala D., Orengo J.C., Abac S., Ruiz A., Laboy J., Flores I. (2010). Patients’ report on how endometriosis affects health, work, and daily life. Fertil. Steril..

[8] Figueira P.G.M., Abrão M.S., Krikun G., Taylor H.S. (2011). Stem cells in endometrium and their role in the pathogenesis of endometriosis. Ann. N. Y. Acad. Sci..

[9] Griffith J.S., Liu Y.-G., Tekmal R.R., Binkley P.A., Holden A.E., Schenken R.S. (2010). Menstrual endometrial cells from women with endometriosis demonstrate increased adherence to peritoneal cells and increased expression of CD44 splice variants. Fertil. Steril..

[10] Goumenou A.G., Matalliotakis I.M., Tzardi M., Fragouli Y.G., Mahutte N.G., Arici A. (2004). Apoptosis and Differential Expression of Apoptosis-Related Proteins in Endometriotic Glandular and Stromal Cells. J. Soc. Gynecol. Investig..

[11] Tosti C., Pinzauti S., Santulli P., Chapron C., Petraglia F. (2015). Pathogenetic Mechanisms of Deep Infiltrating Endometriosis. Reprod. Sci..

[12] Choi J., Jo M., Lee E., Choi D. (2011). The Role of Autophagy in Corpus Luteum Regression in the Rat. Biol. Reprod..

[13] Choi J., Jo M., Lee E., Choi D. (2010). Induction of apoptotic cell death via accumulation of autophagosomes in rat granulosa cells. Fertil. Steril..

[14] Wu Y.C., Wu W.K.K., Li Y., Yu L., Li Z.J., Wong C.C.M., Li H.T., Sung J.J.Y., Cho C.H. (2009). Inhibition of macroautophagy by bafilomycin A1 lowers proliferation and induces apoptosis in colon cancer cells. Biochem. Biophys. Res. Commun..

[15] Klionsky D.J., Emr S.D. (2000). Autophagy as a Regulated Pathway of Cellular Degradation. Science.

[16] Levine B., Klionsky D.J. (2004). Development by Self-Digestion: Molecular Mechanisms and Biological Functions of Autophagy. Dev. Cell.

[17] He C., Klionsky D.J. (2009). Regulation mechanisms and signaling pathways of autophagy. Annu. Rev. Genet..

[18] Mizushima N., Levine B., Cuervo A.M., Klionsky D.J. (2008). Autophagy fights disease through cellular selfdigestion. Nature.

[19] Yang Y.-P., Liang Z.-Q., Gu Z.-L., Qin Z.-H. (2005). Molecular mechanism and regulation of autophagy1. Acta Pharmacol. Sin..

[20] Sun Y., Liu J.-H., Jin L., Pan L., Sui Y.-X., Yang Y., Shi H. (2012). Beclin 1 Influences Cisplatin-Induced Apoptosis in Cervical Cancer CaSki Cells by Mitochondrial Dependent Pathway. Int. J. Gynecol. Cancer.

[21] Ding W.-X., Yin X.-M. (2012). Mitophagy: Mechanisms, pathophysiological roles, and analysis. Biol. Chem..

[22] Boya P., González-Polo R.-A., Casares N., Perfettini J.-L., Dessen P., Larochette N., Métivier D., Meley D., Souquere S., Yoshimori T. (2005). Inhibition of Macroautophagy Triggers Apoptosis. Mol. Cell. Biol..

[23] Boya P., Gonzalez-Polo R.-A., Poncet D., Andreau K., LA Vieira H., Roumier T., Perfettini J.-L., Kroemer G. (2003). Mitochondrial membrane permeabilization is a critical step of lysosome-initiated apoptosis induced by hydroxychloroquine. Oncogene.

[24] Yang H.-L., Mei J., Chang K.-K., Zhou W.-J., Huang L.-Q., Li M.-Q. (2017). Autophagy in endometriosis. Am. J. Transl. Res..

[25] Lockshin R.A., Zakeri Z. (2004). Apoptosis, autophagy, and more. Int. J. Biochem. Cell Biol..

[26] Choi J., Jo M., Lee E., Oh Y.K., Choi D. (2012). The Role of Autophagy in Human Endometrium1. Biol. Reprod..

[27] Mei J., Zhu X.-Y., Jin L.-P., Duan Z.-L., Li D.-J., Li M.-Q. (2015). Estrogen promotes the survival of human secretory phase endometrial stromal cells via CXCL12/CXCR4 up-regulation-mediated autophagy inhibition. Hum. Reprod..

[28] Ruiz A.L.T.G., Rockfield S., Taran N., Haller E., Engelman R.W., Flores I.L., Panina-Bordignon P., Nanjundan M. (2016). Effect of hydroxychloroquine and characterization of autophagy in a mouse model of endometriosis. Cell Death Dis..

[29] Ren X., Wang Y., Xu G., Dai L. (2016). Effect of rapamycin on endometriosis in mice. Exp. Ther. Med..

[30] Siracusa R., D’Amico R., Impellizzeri D., Cordaro M., Peritore A., Gugliandolo E., Crupi R., Salinaro A., Raffone E., Genovese T. (2021). Autophagy and Mitophagy Promotion in a Rat Model of Endometriosis. Int. J. Mol. Sci..

[31] Poulose S.M., Fisher D.R., Larson J., Bielinski D.F., Rimando A.M., Carey A.N., Schauss A.G., Shukitt-Hale B. (2012). Anthocyanin-rich Açai (*Euterpe oleracea* Mart.) Fruit Pulp Fractions Attenuate Inflammatory Stress Signaling in Mouse Brain BV-2 Microglial Cells. J. Agric. Food Chem..

[32] Santos I.B., de Bem G.F., da Costa C.A., de Carvalho L.C.R.M., de Medeiros A.F., Silva D.L.B., Romão M.H., Soares R.D.A., Ognibene D.T., de Moura R.S. (2020). Açaí seed extract prevents the renin-angiotensin system activation, oxidative stress and inflammation in white adipose tissue of high-fat diet–fed mice. Nutr. Res..

[33] Impellizzeri D., D’Amico R., Fusco R., Genovese T., Peritore A.F., Gugliandolo E., Crupi R., Interdonato L., Di Paola D., Di Paola R. (2022). Açai Berry Mitigates Vascular Dementia-Induced Neuropathological Alterations Modulating Nrf-2/Beclin1 Pathways. Cells.

[34] Melo P.S., Massarioli A.P., Lazarini J.G., Soares J.C., Franchin M., Rosalen P.L., de Alencar S.M. (2020). Simulated gastrointestinal digestion of Brazilian açaí seeds affects the content of flavan-3-ol derivatives, and their antioxidant and anti-inflammatory activities. Heliyon.

[35] Rodrigues R.B., Lichtenthäler R., Zimmermann B.F., Papagiannopoulos M., Fabricius H., Marx F., Maia J.G.S., Almeida O. (2006). Total Oxidant Scavenging Capacity of *Euterpe oleracea* Mart. (Açaí) Seeds and Identification of Their Polyphenolic Compounds. J. Agric. Food Chem..

[36] Lee J.Y., Kim N., Choi Y.J., Nam R.H., Lee S., Ham M.H., Suh J.H., Lee H.S., Lee D.H. (2016). Anti-inflammatory and Anti-tumorigenic Effects of Açai Berry in *Helicobacter felis*-infected mice. J. Cancer Prev..

[37] de Moura R.S., Ferreira T.S., Lopes A.A., Pires K.M.P., Nesi R.T., Resende A.C., Souza P.J.C., da Silva A.J.R., Borges R.M., Porto L.C. (2012). Effects of Euterpe oleracea Mart. (AÇAÍ) extract in acute lung inflammation induced by cigarette smoke in the mouse. Phytomedicine.

[38] Kang J., Li Z., Wu T., Jensen G.S., Schauss A.G., Wu X. (2010). Anti-oxidant capacities of flavonoid compounds isolated from acai pulp (*Euterpe oleracea* Mart.). Food Chem..

[39] Arnoso B.J.D.M., Magliaccio F.M., de Araújo C.A., Soares R.D.A., Santos I.B., de Bem G.F., Fernandes-Santos C., Ognibene D.T., de Moura R.S., Resende A.C. (2021). Açaí seed extract (ASE) rich in proanthocyanidins improves cardiovascular remodeling by increasing antioxidant response in obese high-fat diet-fed mice. Chem. Interact..

[40] Bellucci E.R.B., Dos Santos J.M., Carvalho L.T., Borgonovi T.F., Lorenzo J.M., da Silva-Barretto A.C. (2022). Açaí extract powder as natural antioxidant on pork patties during the refrigerated storage. Meat Sci..

[41] da Silva T.V.N., Torres M.F., Sampaio L.A., Hamoy M., Monserrat J.M., Barbas L.A.L. (2021). Dietary Euterpe oleracea Mart. attenuates seizures and damage to lipids in the brain of Colossoma macropomum. Fish Physiol. Biochem..

[42] Kim K.J., Kim Y., Jin S.G., Kim J.Y. (2021). Acai berry extract as a regulator of intestinal inflammation pathways in a Caco-2 and RAW 264.7 co-culture model. J. Food Biochem..

[43] Siracusa R., D’Amico R., Cordaro M., Peritore A., Genovese T., Gugliandolo E., Crupi R., Impellizzeri D., Cuzzocrea S., Fusco R. (2021). The Methyl Ester of 2-Cyano-3,12-Dioxooleana-1,9-Dien-28-Oic Acid Reduces Endometrial Lesions Development by Modulating the NFkB and Nrf2 Pathways. Int. J. Mol. Sci..

[44] Burns K.A., Pearson A.M., Slack J.L., Elaine D., Scribner A.N., Eti N.A., Burney R.O. (2022). Endometriosis in the Mouse: Challenges and Progress Toward a ‘Best Fit’ Murine Model. Front. Physiol..

[45] de Bem G.F., Okinga A., Ognibene D.T., da Costa C.A., Santos I.B., Soares R.A., Silva D.L.B., da Rocha A.P.M., Fernandes J.I., Fraga M.C. (2020). Anxiolytic and antioxidant effects of *Euterpe oleracea* Mart. (açaí) seed extract in adult rat offspring submitted to periodic maternal separation. Appl. Physiol. Nutr. Metab..

[46] Genovese T., Cordaro M., Siracusa R., Impellizzeri D., Caudullo S., Raffone E., Macrí F., Interdonato L., Gugliandolo E., Interlandi C. (2022). Molecular and Biochemical Mechanism of Cannabidiol in the Management of the Inflammatory and Oxidative Processes Associated with Endometriosis. Int. J. Mol. Sci..

[47] Cordaro M., Siracusa R., Fusco R., D’Amico R., Peritore A., Gugliandolo E., Genovese T., Scuto M., Crupi R., Mandalari G. (2020). Cashew (*Anacardium occidentale* L.) Nuts Counteract Oxidative Stress and Inflammation in an Acute Experimental Model of Carrageenan-Induced Paw Edema. Antioxidants.

[48] Di Paola D., Iaria C., Capparucci F., Cordaro M., Crupi R., Siracusa R., D’Amico R., Fusco R., Impellizzeri D., Cuzzocrea S. (2021). Aflatoxin B1 Toxicity in Zebrafish Larva (*Danio rerio*): Protective Role of *Hericium erinaceus*. Toxins.

[49] Cordaro M., Salinaro A.T., Siracusa R., D’Amico R., Impellizzeri D., Scuto M., Ontario M., Interdonato L., Crea R., Fusco R. (2021). Hidrox^®^ and Endometriosis: Biochemical Evaluation of Oxidative Stress and Pain. Antioxidants.

[50] Fusco R., Cordaro M., Siracusa R., Peritore A.F., Gugliandolo E., Genovese T., D’Amico R., Crupi R., Smeriglio A., Mandalari G. (2020). Consumption of *Anacardium occidentale* L. (Cashew Nuts) Inhibits Oxidative Stress through Modulation of the Nrf2/HO−1 and NF-kB Pathways. Molecules.

[51] Di Paola D., Capparucci F., Lanteri G., Crupi R., Marino Y., Franco G.A., Cuzzocrea S., Spanò N., Gugliandolo E., Peritore A.F. (2022). Environmental Toxicity Assessment of Sodium Fluoride and Platinum-Derived Drugs Co-Exposure on Aquatic Organisms. Toxics.

[52] Di Paola D., Iaria C., Capparucci F., Arangia A., Crupi R., Cuzzocrea S., Spanò N., Gugliandolo E., Peritore A.F. (2022). Impact of Mycotoxin Contaminations on Aquatic Organisms: Toxic Effect of Aflatoxin B1 and Fumonisin B1 Mixture. Toxins.

[53] A Zimmerman M., Wilkison S., Qi Q., Chen G., Li P.A. (2020). Mitochondrial dysfunction contributes to Rapamycin-induced apoptosis of Human Glioblastoma Cells—A synergistic effect with Temozolomide. Int. J. Med. Sci..

[54] Fusco R., Cordaro M., Siracusa R., D’Amico R., Genovese T., Gugliandolo E., Peritore A.F., Crupi R., Impellizzeri D., Cuzzocrea S. (2020). Biochemical Evaluation of the Antioxidant Effects of Hydroxytyrosol on Pancreatitis-Associated Gut Injury. Antioxidants.

[55] Di Paola R., Fusco R., Gugliandolo E., D’Amico R., Campolo M., Latteri S., Carughi A., Mandalari G., Cuzzocrea S. (2018). The Antioxidant Activity of Pistachios Reduces Cardiac Tissue Injury of Acute Ischemia/Reperfusion (I/R) in Diabetic Streptozotocin (STZ)-Induced Hyperglycaemic Rats. Front. Pharmacol..

[56] Cordaro M., Siracusa R., D’Amico R., Genovese T., Franco G., Marino Y., Di Paola D., Cuzzocrea S., Impellizzeri D., Di Paola R. (2022). Role of Etanercept and Infliximab on Nociceptive Changes Induced by the Experimental Model of Fibromyalgia. Int. J. Mol. Sci..

[57] Peritore A.F., Crupi R., Scuto M., Gugliandolo E., Siracusa R., Impellizzeri D., Cordaro M., D’Amico R., Fusco R., Di Paola R. (2020). The Role of Annexin A1 and Formyl Peptide Receptor 2/3 Signaling in Chronic Corticosterone-Induced Depression-Like behaviors and Impairment in Hippocampal-Dependent Memory. CNS Neurol. Disord.-Drug Targets.

[58] D’Amico R., Gugliandolo E., Cordaro M., Fusco R., Genovese T., Peritore A.F., Crupi R., Interdonato L., Di Paola D., Cuzzocrea S. (2022). Toxic Effects of Endocrine Disruptor Exposure on Collagen-Induced Arthritis. Biomolecules.

[59] D’Amico R., Gugliandolo E., Siracusa R., Cordaro M., Genovese T., Peritore A.F., Crupi R., Interdonato L., Di Paola D., Cuzzocrea S. (2022). Toxic Exposure to Endocrine Disruptors Worsens Parkinson’s Disease Progression through NRF2/HO-1 Alteration. Biomedicines.

[60] Cordaro M., Fusco R., D’Amico R., Siracusa R., Peritore A., Gugliandolo E., Genovese T., Crupi R., Mandalari G., Cuzzocrea S. (2020). Cashew *(Anacardium occidentale* L.) Nuts Modulate the Nrf2 and NLRP3 Pathways in Pancreas and Lung after Induction of Acute Pancreatitis by Cerulein. Antioxidants.

[61] D’Iglio C., Albano M., Famulari S., Savoca S., Panarello G., Di Paola D., Perdichizzi A., Rinelli P., Lanteri G., Spanò N. (2021). Intra- and interspecific variability among congeneric Pagellus otoliths. Sci. Rep..

[62] Di Paola D., Abbate J.M., Iaria C., Cordaro M., Crupi R., Siracusa R., D’Amico R., Fusco R., Impellizzeri D., Cuzzocrea S. (2022). Environmental Risk Assessment of Dexamethasone Sodium Phosphate and Tocilizumab Mixture in Zebrafish Early Life Stage (*Danio rerio*). Toxics.

[63] Di Paola D., Capparucci F., Abbate J.M., Cordaro M., Crupi R., Siracusa R., D’Amico R., Fusco R., Genovese T., Impellizzeri D. (2022). Environmental Risk Assessment of Oxaliplatin Exposure on Early Life Stages of Zebrafish (*Danio rerio*). Toxics.

[64] D’Amico R., Salinaro A.T., Fusco R., Cordaro M., Impellizzeri D., Scuto M., Ontario M., Dico G.L., Cuzzocrea S., Di Paola R. (2021). *Hericium erinaceus* and *Coriolus versicolor* Modulate Molecular and Biochemical Changes after Traumatic Brain Injury. Antioxidants.

[65] Fusco R., Cordaro M., Siracusa R., Peritore A.F., D’Amico R., Licata P., Crupi R., Gugliandolo E. (2020). Effects of Hydroxytyrosol against Lipopolysaccharide-Induced Inflammation and Oxidative Stress in Bovine Mammary Epithelial Cells: A Natural Therapeutic Tool for Bovine Mastitis. Antioxidants.

[66] Di Paola D., Capparucci F., Lanteri G., Cordaro M., Crupi R., Siracusa R., D’Amico R., Fusco R., Impellizzeri D., Cuzzocrea S. (2021). Combined Toxicity of Xenobiotics Bisphenol A and Heavy Metals on Zebrafish Embryos (*Danio rerio*). Toxics.

[67] D’Amico R., Impellizzeri D., Genovese T., Fusco R., Peritore A.F., Crupi R., Interdonato L., Franco G., Marino Y., Arangia A. (2022). Açai Berry Mitigates Parkinson’s Disease Progression Showing Dopaminergic Neuroprotection via Nrf2-HO1 Pathways. Mol. Neurobiol..

[68] Genovese T., D’Amico R., Fusco R., Impellizzeri D., Peritore A.F., Crupi R., Interdonato L., Gugliandolo E., Cuzzocrea S., Di Paola R. (2022). Açaí (*Euterpe oleraceae* Mart.) Seeds Regulate NF-κB and Nrf2/ARE Pathways Protecting Lung against Acute and Chronic Inflammation. Cell Physiol. Biochem..

[69] Choi J., Jo M., Lee E., Kim H.J., Choi D. (2013). Differential induction of autophagy by mTOR is associated with abnormal apoptosis in ovarian endometriotic cysts. Mol. Hum. Reprod..

[70] Corcelle E.A., Puustinen P., Jäättelä M. (2009). Apoptosis and autophagy: Targeting autophagy signalling in cancer cells—‘Trick or treats’?. FEBS J..

[71] Pant A., Lee I., Lu Z., Rueda B.R., Schink J., Kim J.J. (2012). Inhibition of AKT with the Orally Active Allosteric AKT Inhibitor, MK-2206, Sensitizes Endometrial Cancer Cells to Progestin. PLoS ONE.

[72] Di Tu Q., Jin J., Hu X., Ren Y., Zhao L., He Q. (2020). Curcumin Improves the Renal Autophagy in Rat Experimental Membranous Nephropathy via Regulating the PI3K/AKT/mTOR and Nrf2/HO-1 Signaling Pathways. BioMed Res. Int..

[73] Peritore A., D’Amico R., Siracusa R., Cordaro M., Fusco R., Gugliandolo E., Genovese T., Crupi R., Di Paola R., Cuzzocrea S. (2021). Management of Acute Lung Injury: Palmitoylethanolamide as a New Approach. Int. J. Mol. Sci..

[74] Russell R.C., Yuan H.-X., Guan K.-L. (2013). Autophagy regulation by nutrient signaling. Cell Res..

[75] Rao Y., Perna M.G., Hofmann B., Beier V., Wollert T. (2016). The Atg1–kinase complex tethers Atg9-vesicles to initiate autophagy. Nat. Commun..

[76] Tanida I., Ueno T., Kominami E. (2008). LC3 and Autophagy. Autophagosome and Phagosome.

[77] Komatsu M., Ichimura Y. (2010). MBSJ MCC Young Scientist Award 2009 REVIEW: Selective autophagy regulates various cellular functions. Genes Cells.

[78] Johansen T., Lamark T. (2011). Selective autophagy mediated by autophagic adapter proteins. Autophagy.

[79] Jin S.M., Lazarou M., Wang C., Kane L.A., Narendra D.P., Youle R.J. (2010). Mitochondrial membrane potential regulates PINK1 import and proteolytic destabilization by PARL. J. Cell Biol..

[80] Narendra D.P., Jin S.M., Tanaka A., Suen D.-F., Gautier C.A., Shen J., Cookson M.R., Youle R.J. (2010). PINK1 Is Selectively Stabilized on Impaired Mitochondria to Activate Parkin. PLoS Biol..

[81] Greene A.W., Grenier K., Aguileta M.A., Muise S., Farazifard R., Haque M.E., McBride H.M., Park D.S., Fon E.A. (2012). Mitochondrial processing peptidase regulates PINK1 processing, import and Parkin recruitment. EMBO Rep..

[82] Lee Y., Lee H.-Y., Hanna R.A., Gustafsson Å.B. (2011). Mitochondrial autophagy by Bnip3 involves Drp1-mediated mitochondrial fission and recruitment of Parkin in cardiac myocytes. Am. J. Physiol.-Heart Circ. Physiol..

[83] Siracusa R., D’Amico R., Fusco R., Impellizzeri D., Peritore A.F., Ggliandolo E., Crupi R., Interdonato L., Cordaro M., Cuzzocrea S. (2022). Modulation of NRF-2 Pathway Contributes to the Therapeutic Effects of Boswellia serrata Gum Resin Extract in a Model of Experimental Autoimmune Myocarditis. Antioxidants.

[84] Jin S.M., Youle R.J. (2013). The accumulation of misfolded proteins in the mitochondrial matrix is sensed by PINK1 to induce PARK2/Parkin-mediated mitophagy of polarized mitochondria. Autophagy.

[85] Wanderoy S., Hees J.T., Klesse R., Edlich F., Harbauer A.B. (2020). Kill one or kill the many: Interplay between mitophagy and apoptosis. Biol. Chem..

[86] Cui J., Shi S., Sun X., Cai G., Cui S., Hong Q., Chen X., Bai X.-Y. (2013). Mitochondrial Autophagy Involving Renal Injury and Aging Is Modulated by Caloric Intake in Aged Rat Kidneys. PLoS ONE.

[87] Di Paola D., Natale S., Gugliandolo E., Cordaro M., Crupi R., Siracusa R., D’Amico R., Fusco R., Impellizzeri D., Cuzzocrea S. (2022). Assessment of 2-Pentadecyl-2-oxazoline Role on Lipopolysaccharide-Induced Inflammation on Early Stage Development of Zebrafish (*Danio rerio*). Life.

[88] Di Paola D., Natale S., Iaria C., Cordaro M., Crupi R., Siracusa R., D’Amico R., Fusco R., Impellizzeri D., Cuzzocrea S. (2022). Intestinal Disorder in Zebrafish Larvae (*Danio rerio*): The Protective Action of N-Palmitoylethanolamide-oxazoline. Life.

[89] Di Paola D., Natale S., Iaria C., Crupi R., Cuzzocrea S., Spanò N., Gugliandolo E., Peritore A.F. (2022). Environmental Co-Exposure to Potassium Perchlorate and Cd Caused Toxicity and Thyroid Endocrine Disruption in Zebrafish Embryos and Larvae (*Danio rerio*). Toxics.

[90] Crupi R., Palma E., Siracusa R., Fusco R., Gugliandolo E., Cordaro M., Impellizzeri D., De Caro C., Calzetta L., Cuzzocrea S. (2020). Protective Effect of Hydroxytyrosol Against Oxidative Stress Induced by the Ochratoxin in Kidney Cells: In vitro and in vivo Study. Front. Vet. Sci..

[91] Lu M.-C., Ji J.-A., Jiang Z.-Y., You Q.-D. (2016). The Keap1-Nrf2-ARE Pathway As a Potential Preventive and Therapeutic Target: An Update. Med. Res. Rev..

[92] Fusco R., Salinaro A., Siracusa R., D’Amico R., Impellizzeri D., Scuto M., Ontario M., Crea R., Cordaro M., Cuzzocrea S. (2021). Hidrox^®^ Counteracts Cyclophosphamide-Induced Male Infertility through NRF2 Pathways in a Mouse Model. Antioxidants.

[93] Lee M.-Y., Kim S.H., Oh Y.S., Heo S.-H., Kim K.-H., Chae H.D., Kim C.-H., Kang B.M. (2018). Role of interleukin-32 in the pathogenesis of endometriosis: In vitro, human and transgenic mouse data. Hum. Reprod..

[94] Kroemer G., Levine B. (2008). Autophagic cell death: The story of a misnomer. Nat. Rev. Mol. Cell Biol..

[95] Eisenberg-Lerner A., Bialik S., Simon H.-U., Kimchi A. (2009). Life and death partners: Apoptosis, autophagy and the cross-talk between them. Cell Death Differ..

[96] Pattingre S., Tassa A., Qu X., Garuti R., Liang X.H., Mizushima N., Packer M., Schneider M.D., Levine B. (2005). Bcl-2 Antiapoptotic Proteins Inhibit Beclin 1-Dependent Autophagy. Cell.

[97] Maiuri M.C., Zalckvar E., Kimchi A., Kroemer G. (2007). Self-eating and self-killing: Crosstalk between autophagy and apoptosis. Nat. Rev. Mol. Cell Biol..

[98] Genovese T., Impellizzeri D., D’Amico R., Fusco R., Peritore A.F., Di Paola D., Interdonato L., Gugliandolo E., Crupi R., Di Paola R. (2022). Role of Bevacizumab on Vascular Endothelial Growth Factor in Apolipoprotein E Deficient Mice after Traumatic Brain Injury. Int. J. Mol. Sci..

